# Increased Osteoclastogenesis in Mice Lacking the Carcinoembryonic Antigen-Related Cell Adhesion Molecule 1

**DOI:** 10.1371/journal.pone.0114360

**Published:** 2014-12-09

**Authors:** Timo Heckt, Thomas Bickert, Anke Jeschke, Sebastian Seitz, Jochen Schulze, Wulf D. Ito, Wolfgang Zimmermann, Michael Amling, Thorsten Schinke, Andrea Kristina Horst, Johannes Keller

**Affiliations:** 1 Department of Osteology and Biomechanics, University Medical Center Hamburg-Eppendorf, Hamburg 20246, Germany; 2 Institute of Clinical Chemistry, University Medical Center Hamburg-Eppendorf, Hamburg 20246, Germany; 3 Cardiovascular Center Oberallgäu-Kempten, Im Stillen 3, Immenstadt 87509, Germany; 4 Tumor Immunology Laboratory, LIFE-Center, Klinikum Grosshadern, Ludwig-Maximilians-University Munich, Marchionistraße 15, Munich 81377, Germany; 5 Institute of Experimental Immunology and Hepatology, University Medical Center Hamburg-Eppendorf, Hamburg 20246, Germany; University of Texas Southwestern Medical Center, United States of America

## Abstract

Alterations in bone remodeling are a major public health issue, as therapeutic options for widespread bone disorders such as osteoporosis and tumor-induced osteolysis are still limited. Therefore, a detailed understanding of the regulatory mechanism governing bone cell differentiation in health and disease are of utmost clinical importance. Here we report a novel function of carcinoembryonic antigen-related cell adhesion molecule 1 (CEACAM1), a member of the immunoglobulin superfamily involved in inflammation and tumorigenesis, in the physiologic regulation of bone remodeling. Assessing the expression of all members of the murine Ceacam family in bone tissue and marrow, we found CEACAM1 and CEACAM10 to be differentially expressed in both bone-forming osteoblasts and bone-resorbing osteoclasts. While *Ceacam10*-deficient mice displayed no alteration in structural bone parameters, static histomorphometry demonstrated a reduced trabecular bone mass in mice lacking CEACAM1. Furthermore, cellular and dynamic histomorphometry revealed an increased osteoclast formation in *Ceacam1*-deficient mice, while osteoblast parameters and the bone formation rate remained unchanged. In line with these findings, we detected accelerated osteoclastogenesis in *Ceacam1*-deficient bone marrow cells, while osteoblast differentiation, as determined by mineralization and alkaline phosphatase assays, was not affected. Therefore, our results provide *in vivo* and *in vitro* evidence for a physiologic role of CEACAM1 in the regulation of osteoclastogenesis.

## Introduction

In the healthy organism, bone remodeling is performed by the balanced activity of bone-forming osteoblasts and bone-resorbing osteoclasts, assuring the constant renewal of bone tissue and maintenance of adequate bone stability [1], [2]. In osteoporosis, the most prevalent bone disease worldwide, a relative increase of bone resorption over bone formation occurs, thereby resulting in bone loss and a subsequent increase in fracture risk [3]. As excessive osteoclastogenesis is detrimental not only in osteoporosis, but also tumor-induced osteolysis and Paget's disease of bone [4], [5], the molecular understanding of the processes regulating osteoclast formation and function is of paramount clinical importance.

Osteoclasts represent highly specialized, multinuclear giant cells, which are formed by the fusion of hematopoietic precursor cells from the monocyte/macrophages lineage. The process of osteoclast formation (osteoclastogenesis) depends on two essential cytokines, macrophage colony-stimulating factor (M-CSF) [6], [7] and receptor activator of nuclear factor kappa-B ligand (RANKL) [8], [9], which are produced by bone marrow cells and osteoblasts, respectively. While M-CSF is required for the early differentiation of monocytes and macrophages, RANKL is essential for the subsequent cellular fusion to yield mature and functional osteoclasts. This is best demonstrated by mice lacking RANKL which display osteopetrosis, a condition characterized by the absence of functional osteoclasts and resulting in a marked increase in bone mass with consecutive displacement of bone marrow [10], [11]. Through binding to the receptor activator of nuclear factor κB (RANK), expressed on osteoclasts and their precursors, RANKL activates two key transcription factors, nuclear factor kappa-light-chain-enhancer of activated B-cells (NF-κB) and cytoplasmic calcineurin/nuclear factor of activated t cells (NFATC1), which have been demonstrated to be of crucial importance for osteoclastogenesis [12], [13] Once fully differentiated, osteoclasts express *Acp5* (Tartrate-resistant acid phosphatase) and *Calcr* (Calcitonin receptor) and attach to the bone matrix, which is subsequently resorbed by the secretion of hydrochloric acid and matrix-degrading peptidases [14].

While many systemic and local factors, including endocrine organs, the central nervous system, and mechanical load bearing, have been identified as pivotal regulators of bone turnover [15], [16], recent research has unraveled an unanticipated role of cell adhesion molecules in the regulation of bone cell differentiation and function. For example, vascular cellular adhesion molecule 1, which is expressed on myeloma cells and interacts with integrins mediating osteoclast attachment to bone surface, was shown to tether osteoclast progenitors to accelerate their maturation, thus facilitating tumor-induced osteolysis [17], [18]. Furthermore, it could be demonstrated that the intercellular adhesion molecule-1 provides a high affinity adhesion between osteoblast and osteoclast precursors, thereby enhancing the binding of Rank to membrane-bound Rankl on osteoblasts [19]. Another group of cell-to-cell adhesion molecules that has raised great scientific and clinical interest in recent years are carcinoembryonic antigen-related cell adhesion molecules (CEACAMs), representing a subdivision of the immunoglobulin-related glycoproteins. Apart from functioning as receptors for host-specific bacteria and viruses, CEACAMs have been shown to regulate tissue architecture, cell-to-cell recognition, tumor proliferation, neovascularization and metastasis [20]. However, despite the extensive characterization of CEACAMs in pathologic conditions such as inflammation and cancer, their role in bone remodeling remained unclear to date.

In the present study, we found *Ceacam1* and *Ceacam10* to be expressed in bone marrow and tissue, including osteoblasts and osteoclast precursors. While no alterations in bone remodeling were detected in *Ceacam10*-deficient mice, an osteoporotic bone phenotype due to increased osteoclastogenesis was observed in mice lacking *Ceacam1*. *Ex vivo* assays demonstrated an increased osteoclast formation in bone marrow cultures derived from *Ceacam1*-deficient mice, which was accompanied by an elevated expression of *Nfatc1*, the master transcription factor governing osteoclastogenesis. Taken together, these findings not only provide *in vivo* and *in vitro* evidence for a role of CEACAM1 in the regulation of bone remodeling, they also raise the possibility that pharmacologic targeting of CEACAM1 may be an alternative approach to treat skeletal disorders caused by excessive bone resorption.

## Materials and Methods

### 1. Animals

*Ceacam1*- and *Ceacam10*-deficient animals were generated and genotyped as described previously [21], [22], [23]. All animal experiments were approved by the local animal care committee.

### 2. Skeletal analysis

All mice received dual calcein injections for the determination of the bone formation rate at 9 and 7 days before sacrifice. The lumbar vertebrae were dehydrated and embedded non-decalcified into methyl methacrylate for sectioning. 4 µm-thin sections were stained with toluidine blue or von Kossa/van Gieson procedure as described [24]. Static and cellular histomorphometry was carried out using the OsteoMeasure system (Osteometrics, Decatur, USA) following the guidelines of the American Society of Bone and Mineral Research. Dynamic histomorphometry for the determination of the bone formation rate was performed on non-stained 12 µm-sections. The cortical thickness and the mean diameter of femora were quantified by µCT scanning using a µCT 40 (Scanco Medical).

### 3. Primary osteoblasts

Primary osteoblasts were isolated from bone marrow cells derived from 12 to 18-week-old mice as described [25]. At 80% confluency, cells were differentiated by adding β-glycerophosphate and ascorbic acid. For staining with alizarin red, cells were fixed with 90% ethanol, washed twice with water and incubated with 40 mM alizarin red staining solution (pH 4.2). After washing, the stained cultures were incubated with 10% acetic acid to quantify matrix mineralization. After removing the cellular remnants by centrifugation, the supernatant was neutralized with ammonium hydroxide, and absorption was determined at 405 nm.

### 4. Primary osteoclasts

For the assessment of osteoclastogenesis, the bone marrow from 12 to 18-week-old mice was flushed out of the femora as described previously [25]. At day 2 of differentiation, M-CSF (Peprotech) was added, followed by RANKL (Peprotech) at day 4.

For TRAP-activity staining, cells were washed with PBS and then fixed in cold methanol. After two washing steps with distilled water, the fixed cells were dried before the TRAP-specific substrate naphthol AS-MX phosphate (Sigma-Aldrich) was added. For the quantification of osteoclast formation, the number of TRAP-positive, multinucleated (>3 nuclei/cell) cells per well was determined.

### 5. RAW264.7 cell line

RAW264.7 cells were obtained from ATCC (Wesel, Germany) and cultured in DMEM containing 10% fetal calf serum. For induction of osteoclastogenesis cells were cultured with RANKL for 5 days.

### 6. Expression analysis

Total RNA was isolated from indicated tissues, whole and marrow-flushed bones, or primary bone cells using the RNeasy Minikit (QIAGEN). Subsequently, DNase digestion was performed according to the manufacturer's instructions. Concentration and quality of extracted RNA was measured using an ND-1000 system (NanoDrop Technology). For cDNA synthesis, 0,5–1 µg of RNA was reverse transcribed using cAMV First-Strand Synthesis Kit (Invitrogen) according to the manufacturer's instructions. For semi-quantitative RT-PCR analysis, the following primers were used to amplify fragments of *Ceacam1^a^* (5′-TCAGCACATCTCCACAAAGG-3′ and 5′-CTCTCTGCCGCTGTATGCTT-3′), *Ceacam1/2* (5′-AGCGTCAGGAGGAGCAACTCAA-3′ and 5′-AGAAGAAGGGGCTGAAGTTGGC-3′), *Ceacam2* (5′-GCTATGAAAAGCAGGGCAGA-3′ and 5′-TGAAATTGTCCAGTCAGGACC-3′), *Ceacam9* (5′-CTTAACCTGCTGGAATGCACCCGCCG-3′ and 5′-CAGCTTCTGTTACCGCGGTGCTGTCT-3′), *Ceacam10* (5′-GCTAGATCAAAACTTTGAAATTACTCC-3′ and 5′-ACAGGCATTAGGGTATGATCG-3′), *Ceacam11* (5′-CACAGGAGTTAAACCACTCAAGAA-3′ and 5′-AAACCTGCAGGAGAATATTGTCA-3′), *Ceacam12* (5′-AAGGAGGTAAACTGCTCAAGAT-3′ and 5′-AGTTGAGAAGTAGGATGCTTTC-3′), *Ceacam13* (5′-GGAGCTGCACCGTTCAAGT-3′ and 5′-TGCGTCTTTCTTCTTGACATTG-3′), *Ceacam14* (5′-CCTGGTTCACAGGAGCTAGAGT-3′ and 5′-GGCATCTGAAAGACCCACAA-3′), *Ceacam15* (5′-CCTCTAAAGAAATGCGCTTCTC-3′ and 5′-GACACGCAGGTGAGAATTGA-3′), *Ceacam16* (5′-TCCTGGTGGCCAGTTACATT-3′ and 5′-GCTGCTACAGACGAGACGAA-3′), *Ceacam17* (5′AAACGGCCGATAGACAACGA-3′ and 5′-GAACGGGTCACTATGGAAGG-3′), *Ceacam18* (5′-TGAAGTGGACACTAGCAACG-3′ and 5′-TGCTTAGGAAGGAGCCGTTA-3′), *Ceacam19* (5′CACATCGAGATGATCCCAGA-3′ and 5′-TCCCAATGATGATGGCTACC-3′), *Ceacam20* A1-B1-domain (5′-CAAGCTCACCCTCACAGTCA-3′ and 5′-AAGTTCACGGTGTTGCCTTC-3′), *Ceacam20* B2-cytopl. domain 5 (5′-GATCTGCCTCTGTCCTGGTC-3′ and 5′-TGGGGTGATCTTGCAGTAAA-3′), *Psg17* (5′-GGTACAAAGGGGTGGCAA-3′ and 5′- CAAGCTTGTTAAACACAACTGCT-3′), *Psg30* (5′-CTGCACAAATAACCATTGAATTAGA-3′ and 5′-CTTGACTTGCAAAGGGTGATAA-3′), *Psg31* (5′-CATCCCTTTCTACTTGCTACCAA-3′ and 5′-GCTCAGATTTCTCCTCTGCAATT-3′) and *β–actin* (5′-ATG GAT GAC GAT ATC GCT-3′ and 5′-ATGAGGTAGTCTGTCAGGT-3′) according to the study by Zebauser et al. [26]. Due to a considerable homology *Ceacam1a* and *Ceacam2* were specifically amplified in addition to *Ceacam1/2* in order to detect expression of *Ceacam1*. Quantitative RT-PCR expression analysis was performed using StepOnePlus predesigned TaqMan gene expression assays (Applied Biosystems). *β–actin* or *Gapdh* expression was used as an internal control, as indicated. For western blotting CEACAM1 protein was detected using the polyclonal rabbit anti-CEACAM1 antiserum “P1” after transfer of proteins from an SDS-page onto a PVDF membrane [27].

### 7. Serum analysis

Serum concentrations of bone-specific collagen degradation products (Crosslaps) and OPG were quantified using antibody-based detection kits (#AC-06F1, Immunodiagnostic Systems; #MOP00, R&D Systems; #MTR00, R&D Systems). Mice were fasted for 4 h prior to blood sampling.

### 8. Immunofluorescence

For visualization of the actin cytoskeleton, cells were fixed in Formafix (Pathomed, Germany), followed by permeabilization with ice-cold acetone and rinses in phosphate buffered saline. Subsequently, cells were stained with fluorescein-labelled phalloidin and 4′,6-diamidin-2-phenylindol (DAPI; both Molecular Probes, Germany) at room temperature in the dark (1∶300 in phosphate buffered saline). After washes in phosphate buffered saline, slides were mounted with Vectashield mounting media (Vector Labs, United Kingdom). Pictures were taken on a Leica DM5000B fluorescent microscope (Leica, Germany).

### 9. Statistical analysis

All data were analyzed by two-tailed Student's t test using Excel software. All data are reported as mean ± SD. p<0.05 was considered statistically significant.

## Results

**Figure 1:**
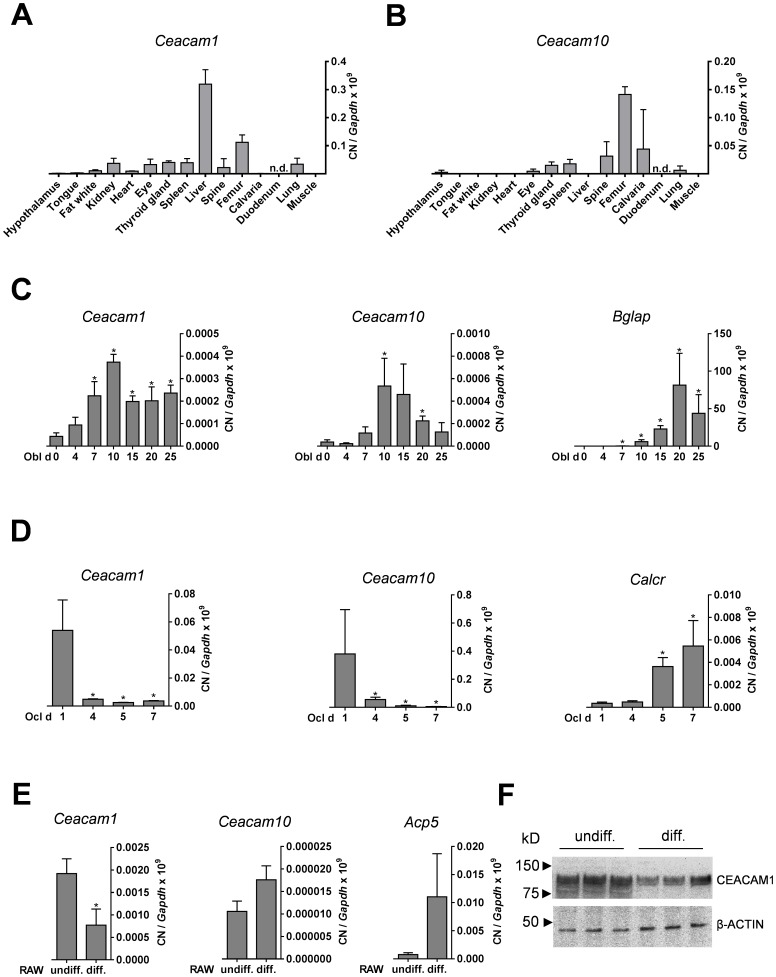
Expression of *Ceacam1* and *Ceacam10* in various tissues and differentiated bone cells. (A, B) qRT-PCR of *Ceacam1* and *Ceacam10* in various tissues. nd  =  not detectable. (C) qRT-PCR of the same genes during osteoblast (Obl) differentiation at the indicated days (d) of differentiation, using *Bglap* expression (OSTEOCALCIN) as a control. (D) qRT-PCR of *Ceacam1* and *Ceacam10* during osteoclast (Ocl) differentiation at the indicated days (d) of differentiation, using *Calcr* expression (CALCITONIN RECEPTOR) as a control. (E) qRT-PCR of *Ceacam1* and *Ceacam10* in undifferentiated (undiff.) and differentiated (diff.) RAW cells, using *Acp5* expression (TRAP) as a control. (F) Western blot showing CEACAM1 expression in cell lysates from undifferentiated and differentiated RAW cells. Arrows indicate size of the nearest marker. All bars represent mean + SD (n≥3 independent experiments). Asterics indicate statistically significant differences compared to day 0 in the case of osteoblasts or day 1 in the case of osteoclasts, respectively (p<0.05).

To elucidate a potential role of CEACAMs in bone remodeling, we monitored the expression of all genes of the CEACAM family in whole bone, bone marrow and marrow-flushed bone. While the expression of most members was not detectable by semi-quantitative PCR, we exclusively found *Ceacam1* and *Ceacam10* expression in bone and bone marrow (S1 Figure). Comparing the expression in various tissues, *Ceacam1* and *Ceacam10* were expressed at comparable levels in bone samples (Fig. 1A). Interestingly, while the expression of *Ceacam1* was highest in liver and femur, *Ceacam10* was expressed at much higher levels in all bone specimens compared to non-skeletal tissues (Fig. 1A). To further characterize the expression of *Ceacam1* and *Ceacam10* in the course of osteoblast and osteoclast formation, we differentiated bone marrow cells into osteoblasts and osteoclasts and performed quantitative RT-PCR. Here we found that the expression of both *Ceacam1* and *Ceacam10* increased during the early stages of osteoblast differentiation, and decreased towards terminal osteoblast differentiation (Fig. 1B). In addition, although *Ceacam1* and *Ceacam10* were both expressed at high levels in undifferentiated bone marrow cultures, their expression markedly decreased during the course of osteoclastogenesis (Fig. 1C). This observation was confirmed in experiments using the macrophage cell line RAW264.7 where we found *Ceacam1* to be differentially expressed during the course of osteoclastogenesis on mRNA (Fig. 1D) and protein level (Fig. 1E).

**Figure 2:**
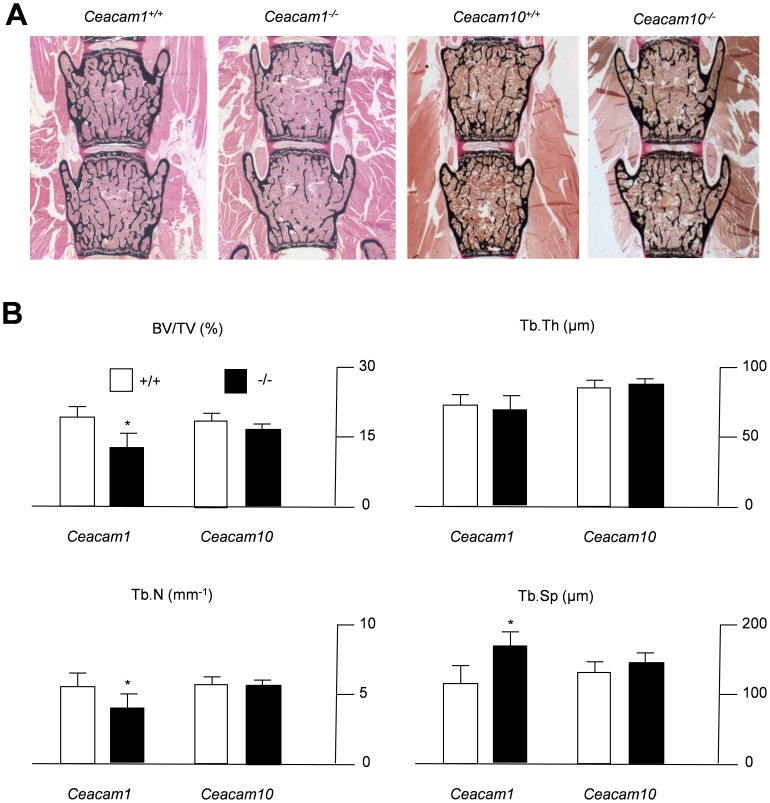
Decreased trabecular bone mass in 6-month-old mice lacking *Ceacam1*. (A) Von Kossa staining of non-decalcified spine sections from controls (*Ceacam1^+/+^*, *Ceacam10^+/+^*) and *Ceacam1*- or *Ceacam10*-deficient mice (*Ceacam1^-/-^*, *Ceacam10^-/-^*). Histomorphometric quantification of the trabecular bone volume (BV/TV, bone volume per tissue volume), trabecular number (Tb.N.), trabecular thickness (Tb.Th.) and trabecular separation (Tb.Sp.). All bars represent mean ± SD (n = 5 mice per group). Asterisks indicate statistically significant differences (p<0.05).

**Figure 3:**
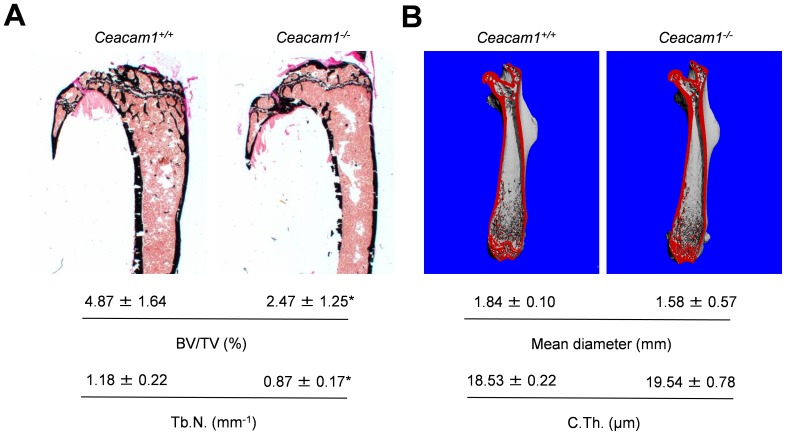
Analysis of long bones derived from 6-month-old mice lacking *Ceacam1*. (A) Von Kossa staining of non-decalcified tibia sections from controls (*Ceacam1^+/+^*) and *Ceacam1*-deficient mice (*Ceacam1^-/-^*) and histomorphometric quantification of the trabecular bone volume (BV/TV) and trabecular number (Tb.N.) below. (B) µCT scanning of femora derived from the same mice. Mean femoral diameter and cortical thickness (C.Th.) are indicated below. All bars represent mean ± SD (n = 5 mice per group). Asterisks indicate statistically significant differences (p<0.05).

As this observation pointed towards a specific role of CEACAM1 and CEACAM10 in the regulation of bone remodeling, we next applied non-decalcified bone histology in mice lacking the respective genes. Von Kossa staining of spine sections from 6-month-old mice demonstrated a reduced bone mass in *Ceacam1*-deficient mice compared to WT controls, whereas no alteration could be detected in mice lacking CEACAM10 (Fig. 2A). These findings were confirmed by static histomorphometry, which demonstrated *Ceacam1*-deficient mice exhibit decreased trabecular bone volume accompanied by a reduction in trabecular number and an increase in trabecular separation (Fig. 2B). In contrast, none of these parameters were altered in mice lacking CEACAM10. In order to analyze whether the observed phenotype is also detectable in younger mice, we additionally performed static histomorphometry of 3-month-old mice. Spine sections of *Ceacam1*-deficient mice were characterized by a significantly reduced trabecular bone mass accompanied by reduced trabecular numbers (S2 Figure), similar to what was observed in 6-month-old animals. In addition, 3-month-old mutant animals displayed a reduced trabecular thickness and increased trabecular separation (S2 Figure). To address the question whether the lack of *Ceacam1* not only affects skeletal architecture in vertebrae, but also long bones, we finally performed histomorphometry of non-decalcified tibia sections and µCT-scanning of femora derived from *Ceacam1*-deficient mice. While we found a decreased trabecular bone volume accompanied by a decrease in trabecular numbers in the tibiae of mutant animals (Fig. 3A), cortical thickness and mean diameter were unaltered in the femora of *Ceacam1*-deficient mice (Fig. 3B).

**Figure 4:**
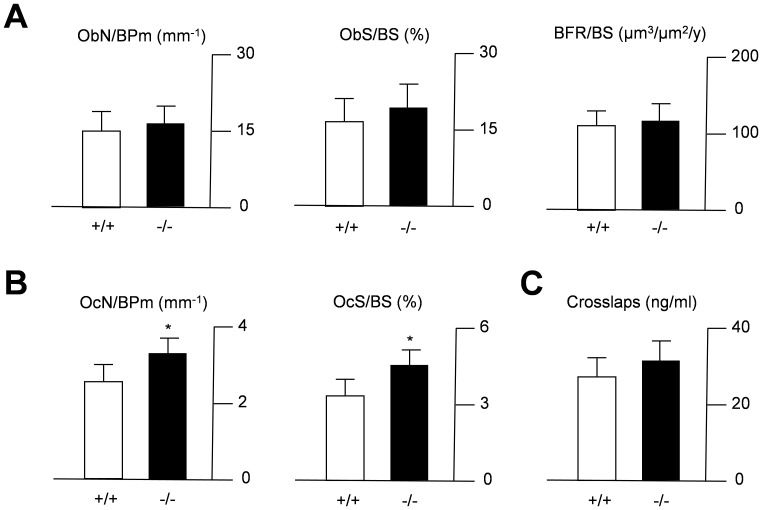
Increased osteoclastogenesis in mice lacking *Ceacam1*. (A) Histomorphometric quantification of the osteoblast number per bone perimeter (ObN/BPm), osteoblast surface per bone surface (ObS/BS) and the bone formation rate per bone surface (BFR/BS). (B) Quantification of the osteoclast number per bone perimeter (OcN/BPm), osteoclast surface per bone surface (OcS/BS). (C) Quantification of serum crosslaps. All bars represent mean ± SD (n = 5 mice per group). Asterisks indicate statistically significant differences (p<0.05).

These findings suggested an important role of CEACAM1 in the regulation of trabecular bone remodeling, while CEACAM10 was found to have no overt effect. Therefore, we focused our further analyses on CEACAM1 and performed a full histomorphometric characterization of the spine sections derived from 6-month-old *Ceacam1*-deficient mice. Cellular histomorphometry using toluidine-blue stained spine sections revealed no alteration in the number of osteoblasts or osteoblast surface, indicating normal osteoblastogenesis in *Ceacam1*-deficient mice (Fig. 4A). Likewise, dynamic histomorphometry following dual calcein labeling demonstrated a normal bone formation rate, ruling out an impaired osteoblast function as the underlying cause of the observed phenotype. In contrast, *Ceacam1*-deficient mice were found to display an increased number of osteoclasts and osteoclast surface, suggesting increased bone resorption (Fig. 4B). Although an absolute increase in collagen degradation products was undetectable (Fig. 4C), normalization of serum crosslaps to the reduced bone mass revealed an elevated net bone resorption in mice lacking CEACAM1 (data not shown).

In order to analyze, whether the observed increase in osteoclastogenesis could be explained by gross alterations in OPG levels, we measured this cytokine in the serum of *Ceacam1*-deficient mice and controls by ELISA. While no changes were detectable in 3-month-old *Ceacam1*-deficient mice, we measured an increased concentration of OPG in 6-month-old mice, pointing towards an age-dependent compensatory regulation *in vivo* in the light of enhanced osteoclastogenesis. This assumption was confirmed *in vitro*, were we detected normal expression of *Tnfsf11* and *Tnfrsf11b*, encoding RANKL and OPG, in primary osteoblasts at day 10 of differentiation (S3 Figure).

**Figure 5:**
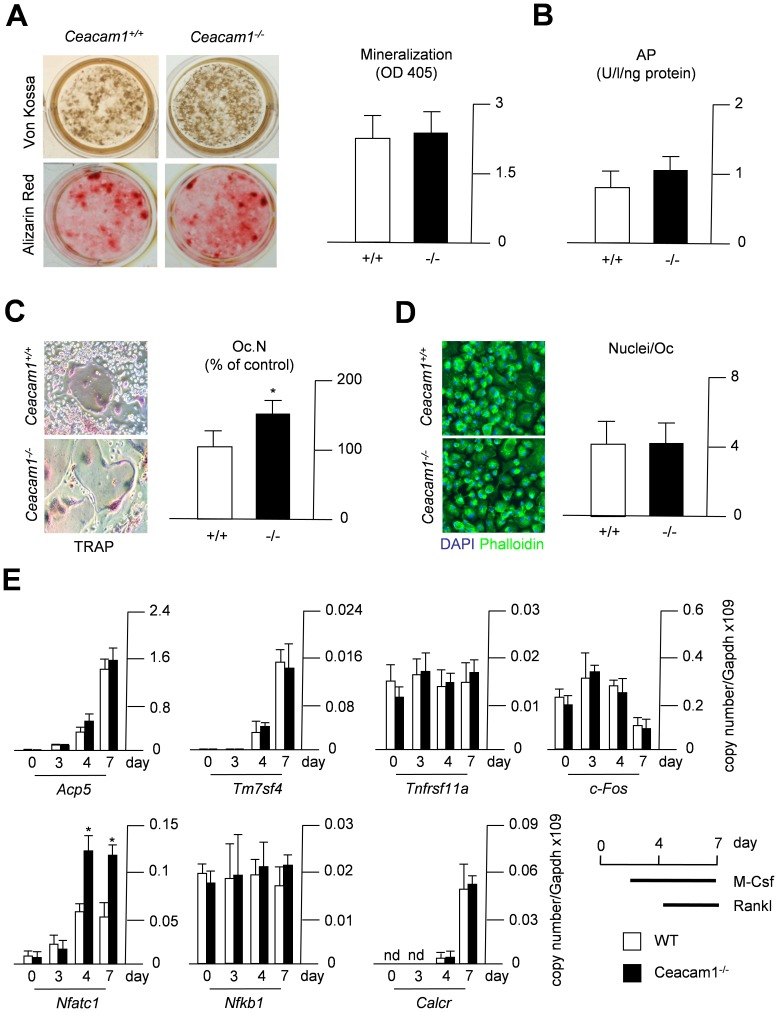
Accelerated osteoclast formation in bone marrow cells lacking *Ceacam1*. (A) Alizarin red and von Kossa staining of mineralized matrix and nodules, respectively, in osteoblast cultures at day 10 of differentiation. The quantification of Alizarin red staining extracellular matrix mineralization is indicated on the right. (B) Quantification of intracellular alkaline phosphatase activity in bone marrow-derived osteoblast cultures at day 10 of differentiation. (C) TRAP activity staining of terminally differentiated osteoclasts. The quantification of TRAP-positive, multinuclear osteoclasts is given on the right. (D) Immunofluorescence using DAPI (nucleus) and Phalloidin (actin) staining of osteoclast cultures at day 7 of differentiation. The quantification of the number of nuclei per osteoclast (Nuclei/Oc) is indicated on the right. (E) qRT-PCR expression analysis of the indicated genes (*Acp5*, Tartrate-resistant acid phosphatase; *Tmf7sf4*, DC-STAMP, dendrocyte-expressed seven transmembrane protein; *NF-κB*, nuclear factor kappa-light-chain-enhancer of activated B-cells; *Nfat1c*, nuclear factor of activated T-cells, cytoplasmic, calcineurin-dependent 1, *Tnfrsf11a*, receptor activator of nuclear factor κB, Rank; *c-Fos*, FBJ osteosarcoma oncogene; and *Calcr*, Calcitonin receptor) at day 0, 3, 4 and 7 of osteoclast differentiation. All bars represent mean ± SD (n = 3 cultures per group). Asterisks indicate statistically significant differences (p<0.05).

Investigating the possibility of cell-autonomous defects in these mice, we next differentiated WT and *Ceacam1*-deficient bone marrow cells into osteoblasts and osteoclasts *ex vivo*. Von Kossa staining at day 10 of differentiation revealed a normal formation of mineralized nodules in *Ceacam1*-deficient osteoblast cultures (Fig. 5A). Likewise, the assessment of alizarin red staining demonstrated regular extracellular matrix mineralization in osteoblasts derived from *Ceacam1*-deficient mice. In addition, normal levels of intracellular alkaline phosphatase activity were measured, ruling out a functional osteoblast defect caused by *Ceacam1*-deficiency (Fig. 5B). In contrast, TRAP-activity staining of *Ceacam1-*deficient bone marrow cells cultured in the presence of M-CSF and RANKL displayed a marked increase in osteoclastogenesis at day 7 of differentiation (Fig. 5C), while no alteration in the number of nuclei per osteoclast could be detected by immunofluorescence (Fig. 5D). To understand this effect on the molecular level, we finally screened for differentially expressed genes essential for osteoclast differentiation and function. On day 0 of differentiation, no significant differences could be measured among the tested genes (Fig. 5E). On day 3 of differentiation, only a tendency towards a reduced expression of *Tm7sf4*, encoding the fusion protein DC-STAMP, could be detected in *Ceacam1*-deficient cultures, whereas the expression of the late osteoclast marker gene *Calcr* was still undetectable in mutant and control cells. In contrast, on day 4 of differentiation we observed a trend towards enhanced expression of *Acp5* and significantly increased expression of *Nfatc1* (Fig. 5E). However, while the increased expression of *Acp5* was only temporarily detectable, we found *Nfatc1* to be overexpressed in *Ceacam1*-deficient osteoclasts again at day 7, pointing towards a crucial role of this key transcription factor in mediating the effects of CEACAM1 on osteoclastogenesis.

## Discussion

The molecular understanding of bone remodeling represents an ongoing clinical challenge, as therapeutic options for skeletal disorders such as osteoporosis and tumor-induced osteolysis caused by excessive osteoclast function remain limited. While the biological actions of CEACAMs have been studied extensively in the context of pathological conditions including inflammation, stroke and cancer [20], [22] a potential role in the regulation of bone turnover has not been sufficiently addressed to date. This is indeed surprising, given the growing body of evidence establishing a link between skeletal integrity and immune or cancer cells not only in basic, but also clinical research. For example, rheumatoid arthritis, a chronic autoimmune disease characterized by joint destruction, is considered to be driven by the secretion of pro-inflammatory cytokines including TNFα and IL-17 from lymphocytes, resulting in excessive activation of osteoclasts [28], [29]. Likewise, expression of IL-1β and inhibitors of Wnt signaling, such as DKK-1, in tumor cells have been reported to be involved in malignant osteolysis through both an activation of osteoclastogenesis and an inhibition of bone formation [30], [31]. Based on their broad array of biological effects in the immune system and tumorigenesis, it was thus important to analyze the role of CEACAMs in the regulation of bone turnover.

Monitoring the expression of all genes encoding the members of the CEACAM family, we could exclusively detect the expression of *Ceacam1* and *Ceacam10* in bone tissue. Although this observation does not necessarily rule out a potential role of other Ceacams in bone remodeling, they pointed towards a specific function of CEACAM1 and CEACAM10 in regulating bone cell function. This notion was further supported by the high cDNA levels of the two encoding genes in bone tissue compared to other organs. Based on the specific expression dynamics of both *Ceacam1* and *Ceacam10* during osteoblast and osteoclast differentiation, we went on to analyze the bone phenotype of the respective animal models. While *Ceacam10*-deficiency was not associated with any alterations in structural bone parameters, static histomorphometry demonstrated a decreased trabecular bone volume in 3- and 6-month old *Ceacam1*-deficient mice. This was indeed an interesting finding, as *Ceacam1*-deficient mice, under basal conditions, have been reported to display no gross phenotypical abnormalities [20]. As mentioned above, while previous studies have primarily focused on the pathophysiologic functions of CEACAM1 in various *in vivo* and *in vitro* disease models, this observation pointed towards a physiologic role of CEACAM1 in the regulation of bone cell activity.

Although *Ceacam1* was differentially expressed during osteoblastogenesis, the cellular and dynamic histomorphometry failed to detect defective osteoblastogenesis or osteoblast function. Surprisingly, increased osteoclast parameters were found in trabecular bone, indicating accelerated osteoclastogenesis in *Ceacam1*-deficient mice. To further characterize this effect at the cellular level, we differentiated bone marrow cells into osteoblasts and osteoclasts. In line with our *in vivo* observations, primary osteoblasts derived from *Ceacam1*-deficient mice displayed normal matrix mineralization and alkaline phosphatase activity *in vitro*. In contrast, although no alteration in the numbers of nuclei per osteoclasts could be detected, *Ceacam1*-deficient bone marrow cells demonstrated an increased osteoclastogenesis when cultured with M-CSF and RANKL. Therefore, it is now possible to conclude that CEACAM1 functions as a negative regulator of osteoclastogenesis *in vivo* and *in vitro*. The fact that we could detect increased levels of serum OPG in 6-month old animals is interesting, however does not explain the observed bone phenotype and increased osteoclastogenesis associated with the lack of CEACAM1. This is supported by the finding that 3-month-old mutant animals displayed a low bone mass phenotype despite normal OPG levels. Furthermore, as we failed to detect differences in the expression of *Tnfsf11* and *Tnfrsf11b* in primary osteoblasts derived from *Ceacam1*-deficient mice, this particular phenomenon is most likely explained by an age-dependent counter regulatory mechanism rather than an intrinsic osteoblast defect.

On the molecular level, we could detect differential expression of *Ceacam1* not only in bone marrow derived osteoclast progenitors, but also in the pure macrophage cell line RAW264.7, providing a potential explanation for the increased osteoclast formation in *Ceacam1*-deficient mice. Since the formation of mature osteoclasts primarily depends on RANKL-induced activation of key transcription factors and the subsequent expression of several osteoclast marker genes, we monitored the expression of *NF-κβ*, *Nfatc1*, *Acp5*, *Tmf7sf4*, *Tnfrsf11a*, *c-Fos*, and *Calcr* during osteoclastogenesis in bone marrow cells derived from WT and *Ceacam1*-deficient mice. While indicators of mature osteoclasts, including *Calcr* and *Acp5*, were found to be expressed at similar or only temporarily elevated levels compared to WT controls, respectively, increased expression of *Nfatc1* in *Ceacam1*-deficient cells was found at day 4 and 7 of osteoclast differentiation. As this coincided with the induction of monocyte/macrophage fusion into mature osteoclasts by the addition of RANKL to these cultures, it appears tempting to speculate that CEACAM1 may modulate osteoclastogenesis through the regulation of NFATC1, the master transcription factor required for osteoclast differentiation. Although a previous study reported a stimulatory effect of a short splice variant of CEACAM1 on the expression of *Nfatc1* in CD4^+^ T cells [32], further research is necessary to elucidate the influence of CEACAM1 on the regulation of this transcription factor specifically in osteoclasts and myeloid-derived cells. This is of particular importance, as the liver-specific inactivation of *Ceacam1*, characterized by hyperinsulinemia and glucose intolerance, results in decreased osteoclastogenesis [33]. Thus, it appears that the impact of global *Ceacam1* deletion, including cells of the osteoclast lineage, is dominant over the effects of hepatic *Ceacam1* expression on bone metabolism.

Taken together, our study reports a novel function of CEACAM1 in bone remodeling. Using *in vivo* and *in vitro* assays, we demonstrate that deficiency of CEACAM1 is associated with a reduced bone mass due to increased osteoclastogenesis, at least in mice. Given its previously reported function in regulating tumor cell differentiation and the immune system, future studies investigating the role of CEACAM1 in pathologic bone conditions, such as tumor-induced osteolysis and inflammation-induced bone loss, will be of crucial importance.

## Supporting Information

S1 Figure **Expression of** ***Ceacam*** **genes in bone tissue and differentiated bone cells.** RT-PCR of the indicated genes in the spine (S), femur (F), flushed femur (FF) and bone marrow (BM) using the same primers as described previously (26).
(TIF)

S2 Figure **Decreased trabecular bone mass in 3-month-old mice lacking** ***Ceacam1*** **.** Von Kossa staining of non-decalcified spine sections from controls (*Ceacam1^+/+^*) and *Ceacam1*-deficient mice (*Ceacam1^-/-^*). Histomorphometric quantification of the trabecular bone volume (BV/TV, bone volume per tissue volume), trabecular number (Tb.N.), trabecular thickness (Tb.Th.) and trabecular separation (Tb.Sp.). All bars represent mean ± SD (n = 5 mice per group). Asterisks indicate statistically significant differences (p<0.05).
(TIF)

S3 Figure **OPG in** ***Ceacam1*** **-deficient mice.** (A) Serum concentrations of OPG in 3- and 6-month old *Ceacam1*-deficient mice. (B) qRT-PCR of *Tnfsf11* and *Tnfrsf11b* encoding RANKL and OPG, respectively, in primary osteoblasts at day 10 of differentiation. All bars represent mean ± SD (n = 5 mice and n = 3 cultures per group, respectively). Asterisks indicate statistically significant differences (p<0.05).
(TIF)

## References

[1] Harada S, Rodan GA (2003) Control of osteoblast function and regulation of bone mass. Nature 423:349–355. 12748654 10.1038/nature01660

[2] Teitelbaum SL, Ross FP (2003) Genetic regulation of osteoclast development and function. Nat Rev Genet 4:638–649. 12897775 10.1038/nrg1122

[3] Zaidi M (2007) Skeletal remodeling in health and disease. Nat Med 7:791–801. 10.1038/nm1593 17618270

[4] Roodman GD (1995) Osteoclast function in Paget's disease and multiple myeloma. Bone 17:57S–61S. 8579899 10.1016/8756-3282(95)00179-h

[5] Goltzman D (2001) Osteolysis and Cancer. J Clin Invest 107:1219–1220. 11375409 10.1172/JCI13073 PMC209307

[6] Kodama H, Yamasaki A, Nose M, Niida S, Ohgame Y, et al (1991) Congenital osteoclast deficiency in osteopetrotic (op/op) mice is cured by injections of macrophage colony-stimulating factor. J Exp Med 173:269–272. 1985123 10.1084/jem.173.1.269 PMC2118769

[7] Dai XM, Ryan GR, Hapel AJ, Dominguez MG, Russell RG, et al (2002) Targeted disruption of the mouse colony-stimulating factor 1 receptor gene results in osteopetrosis, mononuclear phagocyte deficiency, increased primitive progenitor cell frequencies, and reproductive defects. Blood 99:111–120. 11756160 10.1182/blood.v99.1.111

[8] Lacey DL, Timms E, Tan HL, Kelley MJ, Dunstan CR, et al (1998) Osteoprotegerin ligand is a cytokine that regulates osteoclast differentiation and activation. Cell 93:165–176. 9568710 10.1016/s0092-8674(00)81569-x

[9] Yasuda H, Shima N, Nakagawa N, Yamaguchi K, Kinosaki M, et al (1998) Osteoclast differentiation factor is a ligand for osteoprotegerin/osteoclastogenesis-inhibitory factor and is identical to TRANCE/RANKL. Proc Natl Acad Sci USA 95:3597–3602. 9520411 10.1073/pnas.95.7.3597 PMC19881

[10] Kong YY, Yoshida H, Sarosi I, Tan HL, Timms E, et al (1999) OPGL is a key regulator of osteoclastogenesis, lymphocyte development and lymph-node organogenesis. Nature 397:315–323. 9950424 10.1038/16852

[11] Kim N, Odgren PR, Kim DK, Marks SC Jr, Choi Y (2000) Diverse roles of the tumor necrosis factor family member TRANCE in skeletal physiology revealed by TRANCE deficiency and partial rescue by a lymphocyte-expressed TRANCE transgene. Proc Natl Acad Sci USA 97:10905–10910. 10984520 10.1073/pnas.200294797 PMC27122

[12] Matsuo K, Galson DL, Zhao C, Peng L, Laplace C, et al (2004) Nuclear factor of activated T-cells (NFAT) rescues osteoclastogenesis in precursors lacking c-Fos. J Biol Chem. 279:26475–26480. 15073183 10.1074/jbc.M313973200

[13] Takayanagi H, Kim S, Koga T, Nishina H, Isshiki M, et al (2002) Induction and Activation of the Transcription Factor NFATc1 (NFAT2) Integrate RANKL Signaling in Terminal Differentiation of Osteoclasts. Dev Cell 3:889–901. 12479813 10.1016/s1534-5807(02)00369-6

[14] Boyle WJ, Simonet WS, Lacey DL (2003) Osteoclast differentiation and activation. Nature 423:337–342. 12748652 10.1038/nature01658

[15] Rodan GA (1998) Bone homeostasis. Proc Natl Acad Sci U S A 95:13361–13362. 9811806 10.1073/pnas.95.23.13361 PMC33917

[16] Turner CH (1998) Three rules for bone adaptation to mechanical stimuli. Bone 23:339–409. 10.1016/s8756-3282(98)00118-5 9823445

[17] Michigami T, Shimizu N, Williams PJ, Niewolna M, Dallas SL, et al (2000) Cell-cell contact between marrow stromal cells and myeloma cells via VCAM-1 and alpha(4)beta(1)-integrin enhances production of osteoclast-stimulating activity. Blood 96:1953–1960. 10961900

[18] Pearse RN, Sordillo EM, Yaccoby S, Wong BR, Liau DF, et al (2001) Multiple myeloma disrupts the TRANCE/osteoprotegerin cytokine axis to trigger bone destruction and promote tumor progression. Proc Natl Acad Sci U S A 98:11581–11586. 11562486 10.1073/pnas.201394498 PMC58772

[19] Tanaka Y, Maruo A, Fujii K, Nomi M, Nakamura T, et al (2000) Intercellular adhesion molecule 1 discriminates functionally different populations of human osteoblasts: characteristic involvement of cell cycle regulators. J Bone Miner Res. 15:1912–1923. 11028443 10.1359/jbmr.2000.15.10.1912

[20] Kuespert K, Pils S, Hauck CR (2006) CEACAMs: their role in physiology and pathophysiology. Curr Opin Cell Biol 18:565–571. 16919437 10.1016/j.ceb.2006.08.008 PMC7127089

[21] Bickert T, Marshall RP, Zhang Z, Ludewig P, Binder M, et al (2012) Acceleration of collateral development by carcinoembryonic antigen-related cell adhesion molecule 1 expression on CD11b/^+^Gr-1^+^ myeloid cells–brief report. Arterioscler Thromb Vasc Biol 32:2566–2568. 22962327 10.1161/ATVBAHA.112.300015

[22] Ludewig P, Sedlacik J, Gelderblom M, Bernreuther C, Korkusuz Y, et al (2013) Carcinoembryonic Antigen-Related Cell Adhesion Molecule 1 Inhibits MMP-9-Mediated Blood-Brain-Barrier Breakdown in a Mouse Model for Ischemic Stroke. Circ Res 113:1013–1022. 23780386 10.1161/CIRCRESAHA.113.301207

[23] Finkenzeller D, Fischer B, Lutz S, Schrewe H, Shimizu T, et al (2003) Carcinoembryonic antigen-related cell adhesion molecule 10 expressed specifically early in pregnancy in the decidua is dispensable for normal murine development. Mol Cell Biol 23:272–279. 12482980 10.1128/MCB.23.1.272-279.2003 PMC140660

[24] Albers J, Schulze J, Beil FT, Gebauer M, Baranowsky A, et al (2011) Control of bone formation by the serpentine receptor Frizzled-9. J Cell Biol 192:1057–1072. 21402791 10.1083/jcb.201008012 PMC3063134

[25] Albers J, Keller J, Baranowsky A, Beil FT, Catala-Lehnen P, et al (2013) Canonical Wnt signaling inhibits osteoclastogenesis independent of osteoprotegerin. J Cell Biol 200:537–549. 23401003 10.1083/jcb.201207142 PMC3575535

[26] Zebhauser R, Kammerer R, Eisenried A, McLellan A, Moore T, et al (2005) Identification of a novel group of evolutionarily conserved members within the rapidly diverging murine *Cea* family. Genomics 86:566–580. 16139472 10.1016/j.ygeno.2005.07.008

[27] Horst AK, Bickert T, Brewig N, Ludewig P, van Rooijen N, et al (2009) CEACAM1+ myeloid cells control angiogenesis in inflammation. Blood 113:6726–6736. 19273835 10.1182/blood-2008-10-184556

[28] Brennan FM, McInnes IB (2008) Evidence that cytokines play a role in rheumatoid arthritis. J Clin Invest 118:3537–3545. 18982160 10.1172/JCI36389 PMC2575731

[29] Kotake S, Udagawa N, Takahashi N, Matsuzaki K, Itoh K, et al (1999) IL-17 in synovial fluids from patients with rheumatoid arthritis is a potent stimulator of osteoclastogenesis. J Clin Invest 103:1345–1352. 10225978 10.1172/JCI5703 PMC408356

[30] Tian E, Zhan F, Walker R, Rasmussen E, Ma Y, et al (2003) The role of the Wnt-signaling antagonist DKK1 in the development of osteolytic lesions in multiple myeloma. N Engl J Med 349:2483–2494. 14695408 10.1056/NEJMoa030847

[31] Lust JA, Donovan KA (1999) The role of interleukin-1 beta in the pathogenesis of multiple myeloma. Hematol Oncol Clin North Am 13:1117–1125. 10626139 10.1016/s0889-8588(05)70115-5

[32] Chen L, Chen Z, Baker K, Halvorsen EM, da Cunha AP, et al (2012) The short isoform of the CEACAM1 receptor in intestinal T cells regulates mucosal immunity and homeostasis via Tfh cell induction. Immunity 37:930–946. 23123061 10.1016/j.immuni.2012.07.016 PMC3516394

[33] Huang S, Kaw M, Harris MT, Ebraheim N, McInerney MF, et al (2010) Decreased osteoclastogenesis and high bone mass in mice with impaired insulin clearance due to liver-specific inactivation to CEACAM1. Bone 46:1138–1145. 20044046 10.1016/j.bone.2009.12.020 PMC2862391

