# Supplementary figures and images for: Increased Osteoclastogenesis in Mice Lacking the Carcinoembryonic Antigen-Related Cell Adhesion Molecule 1
Source: PLoS One. 2014 Dec 9;9(12):e114360. doi: 10.1371/journal.pone.0114360 (PMC4260834; doi:10.1371/journal.pone.0114360)

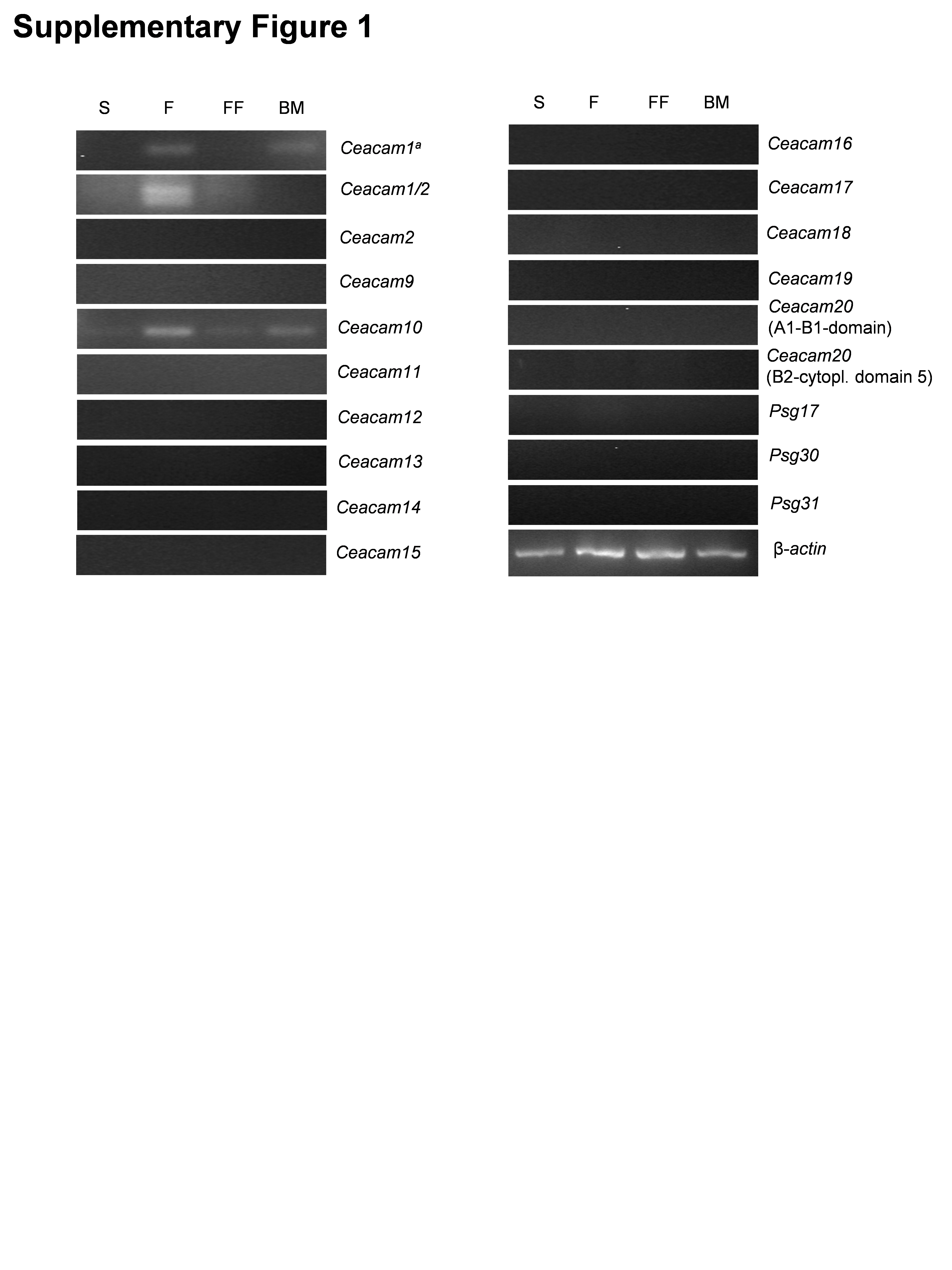

Supplement: S1 Figure — Expression of Ceacam genes in bone tissue and differentiated bone cells. RT-PCR of the indicated genes in the spine (S), femur (F), flushed femur (FF) and bone marrow (BM) using the same primers as described previously (26). (TIF) [file pone.0114360.s001.tif]

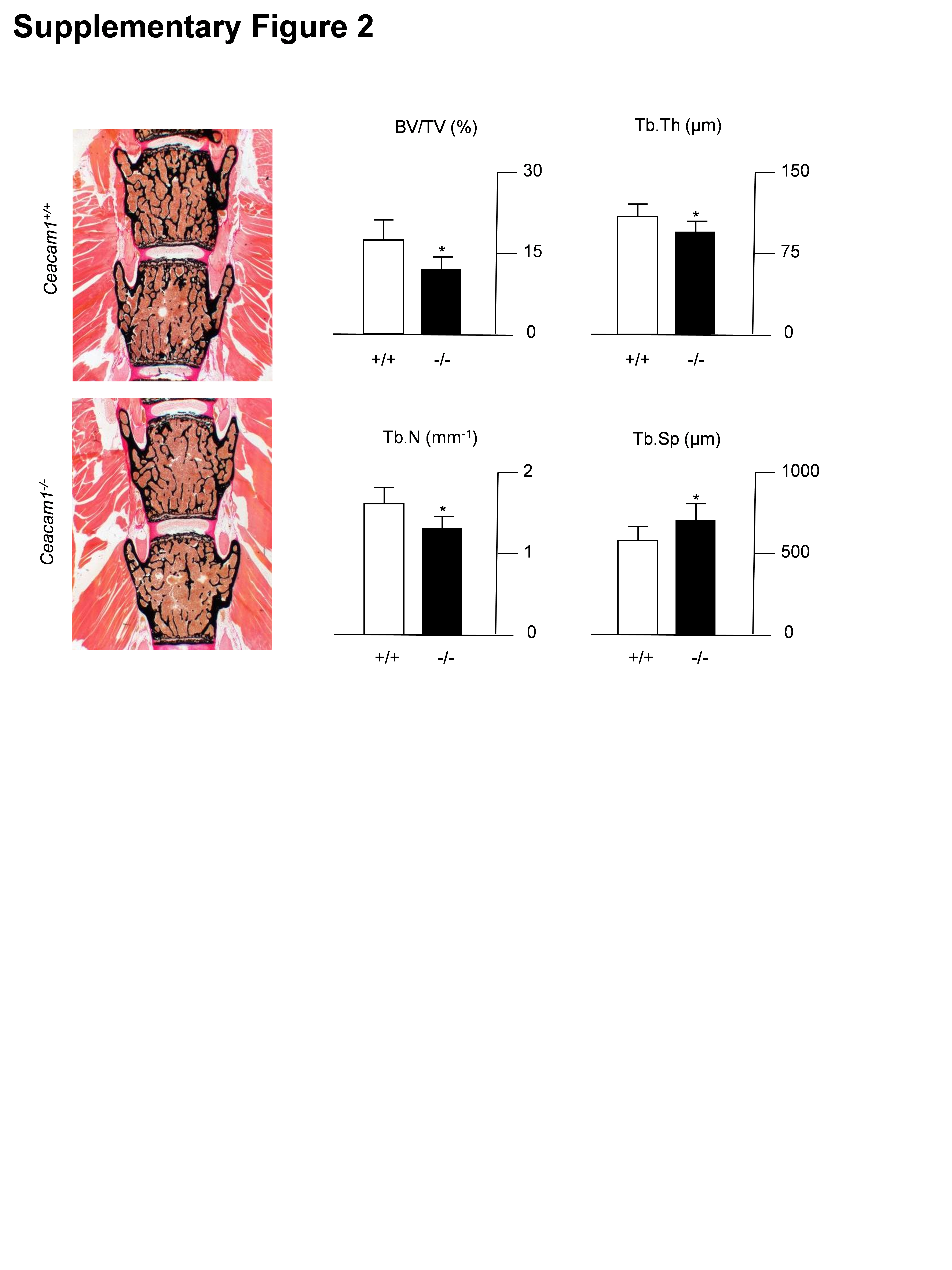

Supplement: S2 Figure — Decreased trabecular bone mass in 3-month-old mice lacking Ceacam1 . Von Kossa staining of non-decalcified spine sections from controls (Ceacam1+/+) and Ceacam1-deficient mice (Ceacam1-/-). Histomorphometric quantification of the trabecular bone volume (BV/TV, bone volume per tissue volume), trabecular number (Tb.N.), trabecular thickness (Tb.Th.) and trabecular separation (Tb.Sp.). All bars represent mean ± SD (n = 5 mice per group). Asterisks indicate statistically significant differences (p<0.05). (TIF) [file pone.0114360.s002.tif]

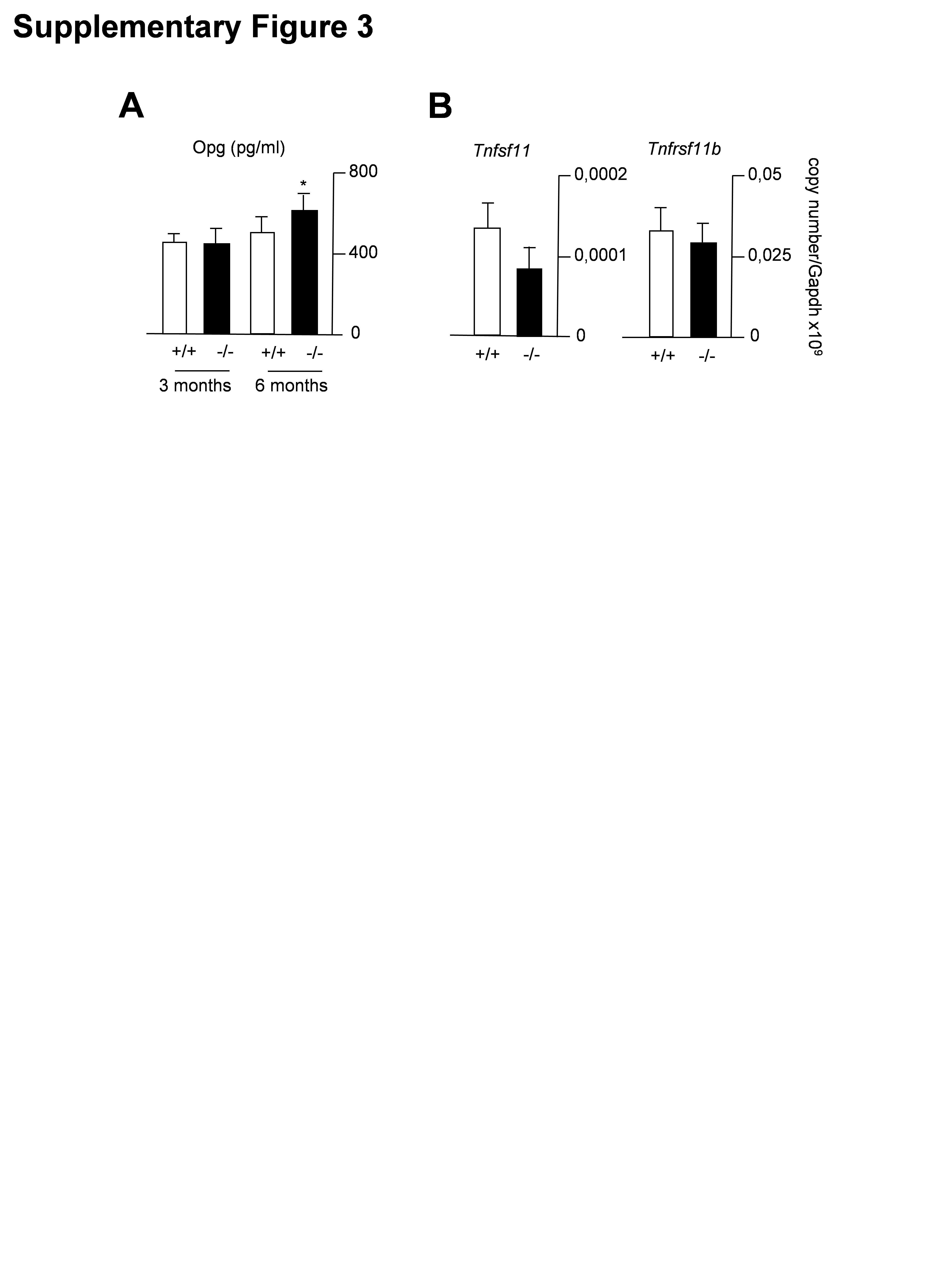

Supplement: S3 Figure — OPG in Ceacam1 -deficient mice. (A) Serum concentrations of OPG in 3- and 6-month old Ceacam1-deficient mice. (B) qRT-PCR of Tnfsf11 and Tnfrsf11b encoding RANKL and OPG, respectively, in primary osteoblasts at day 10 of differentiation. All bars represent mean ± SD (n = 5 mice and n = 3 cultures per group, respectively). Asterisks indicate statistically significant differences (p<0.05). (TIF) [file pone.0114360.s003.tif]
